# Clinical correlation of SARS-CoV-2 wastewater passive sampling in long-term care facilities and wastewater treatment plants

**DOI:** 10.1016/j.envadv.2025.100635

**Published:** 2025-04-19

**Authors:** William Strike, Alexus Rockward, Blazan Mijatovic, Ann Noble, Cullen Olsson, Soroosh Torabi, Mohammad Dehghan Banadaki, Reuben Adatorwovor, James Keck, Scott Berry

**Affiliations:** a Department of Biomedical Engineering, University of Kentucky, 522 Robotics and Manufacturing Building, Lexington, KY 40506-0108, United States; b Department of Mechanical and Aerospace Engineering, University of Kentucky, 151 Ralph G. Anderson Building, Lexington, KY 40506-0503, United States; c Department of Biostatistics, University of Kentucky, 111 Washington Avenue, Lexington, KY 40536, United States; d Department of Family and Community Medicine, University of Kentucky, 2159 Harrodsburg Road, Lexington, KY 40504, United States

**Keywords:** Moore swab, Passive sampling, Wastewater-based epidemiology (WBE), SARS-CoV-2, Long-term care facilities, Clinical correlation

## Abstract

Wastewater-based epidemiology (WBE) is a promising tool for improving health outcomes through early detection and cost-effective pathogen surveillance. Long-term care facilities (LTCFs) serve and employ vulnerable populations that may particularly benefit from the use of WBE, but financial and technical costs associated with standard sampling methods limit the feasibility of WBE in the LTCF setting. In this work, we used passive sampling to simplify the wastewater analysis process and compared its performance to the standard composite sampling method. Moore swabs and automatic composite samplers were used concurrently to sample wastewater from two LTCFs, and samples were analyzed for SARS-CoV-2 concentration. Passive sampling relies on an unknown volume of wastewater flowing through a cotton material, which complicates back calculations of pathogen concentration. We chose to calculate analyte concentrations based on the squeezed eluent from the cotton swab, which is practical for temporal analysis. Across all samples, passive and composite sampling performed similarly for SARS-CoV-2 detection and mean concentration. However, we observed a sensitivity advantage at low SARS-CoV-2 concentrations (<180 gc/mL) when using passive sampling. Furthermore, SARS-CoV-2 wastewater concentrations obtained via passive sampling correlated with the reported clinical cases, with wastewater concentration leading reported clinical cases by an average of 4 days. Passive and composite sampling were also performed at a wastewater treatment plant (WWTP) to examine the effects of facility type on sampling performance. To our knowledge, this is the first work performing a comparative analysis at both facility- and community-scale locations. Passive sampling yielded significantly higher SARS-CoV-2 and fecal load biomarkers than composite sampling at WWTPs, illustrating an important difference between LTCF samples and WWTP samples.

## 1. Introduction

During the COVID-19 pandemic, wastewater-based epidemiology (WBE) emerged as a means of surveilling the prevalence of a pathogen at both the building/facility level (e.g., dormitories, long-term care facilities) and the community level. WBE measures biomarkers in wastewater as a means of assessing the health of the population that contribute to the downstream wastewater infrastructure. The proportion of a biomarker present in the sample is known to be related to its presence in the associated population, enabling the estimation of the number of cases within that population. This environmental surveillance tool has been successfully applied for monitoring and detection of COVID-19 outbreaks in various contexts (Karthikeyan et al., 2021; Medema et al., 2020; Wu et al., 2022; Mao et al., 2020; Strike et al., 2022). Importantly, WBE may capture mildly symptomatic and asymptomatic patients, who are less likely to undergo testing or seek treatment from a healthcare facility and are therefore not captured by the clinical data (Centers for Disease Control and Prevention, 2021; Coronavirus Pandemic (COVID-19), 2020). Moreover, as at-home testing rates increase, a growing proportion of positive cases are not reported to public health authorities, which can further obscure the epidemiological patterns in a community and WBE may provide a less biased assessment of community disease trends (Rader et al., 2022).

While WBE has gained significant traction in many settings, the complexity of traditional WBE workflows has limited impact in remote and rural settings as well as in individual facilities that encompass a smaller number of people. Typical WBE workflows require specialized equipment and technical expertise, such as ultrafiltration and ultracentrifugation, for reliable detection of SARS-CoV-2 (World Health Organization, 2003). While wastewater surveillance has been deployed successfully in some resource-limited settings (Capone et al., 2022; Hovi et al., 2012), the equipment and personnel requirements of traditional WBE protocols can erect substantial financial and logistic barriers to broader deployment in middle- and low-income regions (Mao et al., 2020; Street et al., 2020). For sample collection, a large programmable pump and storage system known as an automatic sampler (or autosampler) is commonly used to collect time-averaged samples from municipal wastewater systems and treatment plants (Zuccato et al., 2008; Graham et al., 2021). This has the advantage of providing automated, semi-continuous sample collection and is well-suited for large wastewater treatment plants (WWTPs) with existing infrastructure to facilitate sampling. However, autosamplers are expensive and require skilled personnel for operation and maintenance, limiting feasibility in regions that lack sufficient human and financial capital. Due to such limitations, different sample collection techniques are necessary to unlock the full potential of WBE globally.

Grab and passive sampling techniques are often used in less developed regions, where much of the sewage is discharged into open-air waterways or pit latrines. Grab sampling, in which a volume of wastewater is sampled at a particular time point, enables sample collection without advanced equipment or technical skill. However, wastewater is subject to temporal heterogeneity in composition due to the intermittent introduction of fecal material into the catchment area, particularly at the facility level (i.e., single-timepoint sampling at the outflow of a single building may fail to collect the feces of an infected individual). Because a grab sample represents a single time point, the resulting data is more variable and is generally less sensitive when compared to other sampling methods (Rafiee et al., 2021; Wang et al., 2022). For such reasons, continuous and semi-continuous samples, such as composite samples, are preferred when analyzing wastewater. Passive sampling techniques combine the cost-effectiveness and simplicity of grab sampling with the potential for continuous sample collection. Passive samplers (often termed Moore swabs) are typically comprised of an absorbent material (e.g., gauze, tampon) that may be protected by some porous housing (e. g., a perforated plastic bottle) and is suspended by string within a wastewater flow, such that liquid continuously perfuses the absorbent material. Passive sampling tools leverage the structural and intrinsic properties of materials to collect samples through physical and/or electrostatic interactions with substances in wastewater (Hayes et al., 2021; Bivins et al., 2022; Jones et al., 2022). This sampling method does not require specialized training or specialized equipment, and the cost is several orders of magnitude lower than that of an autosampler (i.e., the autosampler used in this study costs ~$10k, while the passive sampling setup costs ~$3). These advantages make passive sampling an attractive alternative to composite sampling for low-resource settings.

While the high cost of composite sampling may be justifiable when sampling a large population (e.g., at a municipal wastewater treatment plant), WBE also has utility at the facility level (i.e., a single building housing tens to hundreds of individuals). An important use case for facility-level WBE involves testing at long-term care facilities (LTCFs), which harbor highly vulnerable populations (Lau-Ng et al., 2020; Chidambaram, 2022; Levin et al., 2022). Unfortunately, given that an autosampler device will be required for each facility, WBE becomes increasingly costly and impractical. Alternatively, passive sampling has been used to monitor several infectious diseases in various settings (Sikorski and Levine, 2020), and has demonstrated excellent potential as a simple sampling alternative to grab and composite sampling methods. It is easy and inexpensive to deploy and has been successfully implemented for the capture and analysis of SARS-CoV-2 in wastewater at multiple catchment levels (Corchis-Scott et al., 2021; Liu et al., 2022; Cha et al., 2023). For passive sampling to be a viable alternative in this setting, benchmarking against gold standard composite sampling must demonstrate acceptable analytical sensitivity (Bivins et al., 2022; Cha et al., 2023; Schang et al., 2021; Wilson et al., 2022; Mejías-Molina et al., 2023). In this work, we utilized passive sampling to produce a simplified, low-resource workflow suitable for use at the facility level and compared the performance of passive and composite sampling methods at two long-term care facilities. While most passive sampling methods have been deployed at the community level (Bivins et al., 2022; Cha et al., 2023; Mejías-Molina et al., 2023), facility-level surveillance is appealing in highly vulnerable populations (e.g., LTCF residents). However, moving testing “more upstream” may reduce the level of wastewater homogenization, subsequently altering passive swab capture efficiency. In response, equivalent studies are reported here, which compare the performance of passive and composite sampling at both facility- and community-level sewersheds. Additionally, performance of passive sampling at facility-level LTCFs was compared with performance at WWTPs, given that these samples may differ in composition (e.g., effluent from LTCFs may have different concentrations of cleaning products or other chemicals that may affect pathogens, influent from WWTPs may be more homogenized due to the mixing and shearing encountered during transport through the sewer system).

## 2. Methods

### 2.1. Site selection

Two long-term care facilities (LTCFs) in Lexington, Kentucky were selected for this study, which included both composite and passive sampling between December 8, 2021 and January 4, 2022. A gap in sample collection occurred from December 17, 2021 to January 3, 2022 due to holiday staffing limitations. Additionally, seven samples were collected from a WWTP in Eastern Kentucky, using both passive and composite sampling from September 6, 2022 to October 18, 2022.

**Fig. 1.**
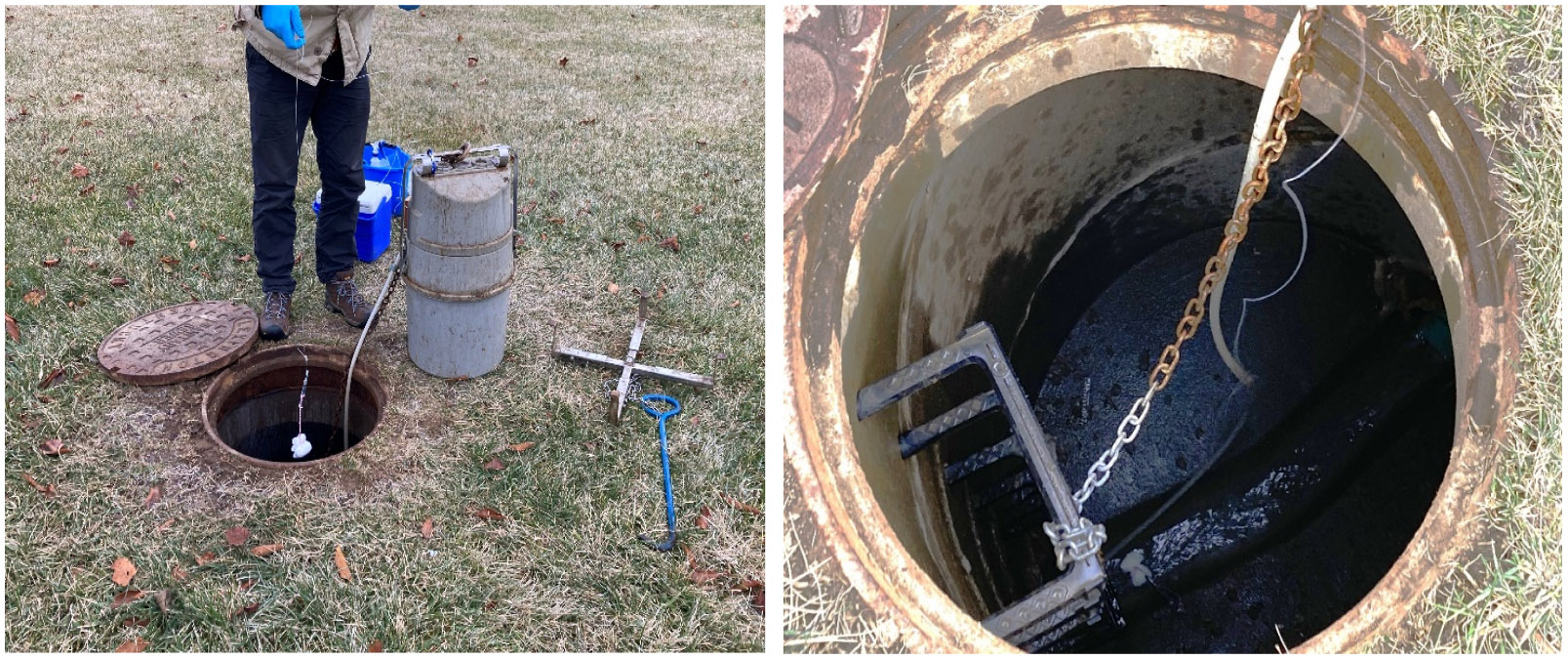
Sampling systems at nursing home location. Left: Technician installing passive sampler in LTCF manhole with composite sampler nearby (grey cylinder). Right: Contrast-adjusted image (to improve visibility in manhole) of passive sampler placement within the effluent channel.

A total of 53 paired samples were collected and processed during the collection period. An automatic composite sampler (Teledyne ISCO, Lincoln, NE) was installed at manholes directly downstream of the LTCF, as shown in Fig. 1. The autosampler was programmed to collect 100 mL of wastewater from the channel every 20 min within a 24-h sampling frame into a 10 L polypropylene collection jug lined with plastic, resulting in a total sample volume of 7.2 L. The exterior of the collection jug was lined with ice to mitigate RNA degradation in the sample. Following sample collection, 250 mL of the collected composite sample was transferred into a polypropylene bottle and stored in a cooler packed with ice during transport to a laboratory at University of Kentucky.

To produce a compact passive sampler, five and a half 4”x 4” cotton gauze sponges were stacked, folded twice, and secured about their midpoint with a cotton string. At the beginning of the 24-h sampling period, a passive sampler was placed adjacent to the inlet tubing of the autosampler in each effluent channel. Nylon line was secured about the bracket supporting the autosampler in the manhole, and an eye-hook turnbuckle was attached to function as both a weight for the line and a connecting point to allow for rapid exchange of the swab. At the end of the sampling period, the passive sampler was removed from the turnbuckle and placed in a resealable plastic bag. The plastic bag was then transported on ice to a laboratory at the University of Kentucky for processing. The passive sample was obtained from the swab by squeezing wastewater from the swab into a corner of the plastic bag. The expressed sample was then poured into a 50 mL conical tube and stored at 4°C in a refrigerator.

### 2.2. Sample extraction

All samples were processed using an Exclusion-based Sample Preparation (ESP) extraction workflow, as described in Strike et al. (2022). Briefly, 20 μL of a 1:1 mixture of SeraSil paramagnetic beads (400 nm and 700 nm, Cytiva) and 375 μL in-house lysis buffer (4 M guanidinium thiocyanate (Thermo Fisher), 10 mM 4-morpholineethanesulfonic acid (MES) sodium salt (Sigma Aldrich), 50 % absolute ethanol) were added to 250 μL aliquots of wastewater sample and incubated at 50°C for 20 min with intermittent mixing to maintain bead suspension. The sample solutions were tumbled for 20 min at low speed, after which the beads were separated out of the solution using a magnetic separation rack, and the supernatant was discarded. The beads were then resuspended in 500 μL Wash Buffer 1 (WB1; 1 M guanidinium thiocyanate (Thermo Fisher), 10 mM Tris buffer pH=8 (Thermo Fisher), and 1 % Tween^®^ 20 solution (Sigma-Aldrich)) and transferred to a plate on the Extractman^®^ platform (Gilson, Inc.) for streamlined washing. This device magnetically manipulates the beads through liquid and air interfaces to limit contaminate carryover between washing steps. The beads were washed twice in WB1, twice in Wash Buffer 2 (WB2; 10 mM Tris buffer pH=8 (Thermo Fisher) and 80 % ethanol/water) and transferred in 110 μL nuclease-free water. After pipette resuspension, the solution was transferred onto a hot plate for 20 min at 70°C to promote elution from the beads. The beads were magnetically removed from solution, and the eluate was transferred into a new microtube for RT-qPCR analysis. All extracted RNA samples were stored at −80°C.

### 2.3. RT-qPCR analysis

All samples were analyzed for the presence of SARS-CoV-2 and crAssphage viruses. RT-qPCR analysis of samples was performed on eight sample replicates using the SARS-CoV-2 N1 gene primers and probe published in Vogels et al. (2020) (forward primer: 5’-GACCCCAAAATCAGCGAAAT-3’, reverse primer: 5’-TCTGGTTACTGCCAGTTGAATCTG-3’, probe: 5’-[FAM] ACCCCGCATTACGTTTGGTGGACC [MGB]–3’, total amplicon = 72 bp). Fecal load was determined by quantifying crAssphage, a DNA bacteriophage commonly found in human feces(31, 32). We measured crAssphage DNA concentrations in two replicates from each wastewater composite sample. We used the same processes for nucleic acid extraction, purification, and quantification as used for SARS-CoV-2. CrAssphage primers and probes published in Stachler et al. (2017) were used for RT-qPCR analysis (forward primer: 5’- CAGAAGTACAAACTCCTAAAAAACGTAGAG-3’, reverse primer: 5’-GATGACCAATAAACAAGCCATTAGC-3’, probe: 5’-[FAM] AATAACGATTTACGTGATGTAAC [MGB]–3’). The 20uL RT-qPCR reaction volume consisted of 5 μL of TaqMan^™^ 4X Fast Virus 1-Step Master Mix (Applied Biosystems), 1 μL of primer and probe (Thermo Fisher, synthesized at a working concentration of 20X, where 1X primer concentration = 900 nM, probe concentration = 250 nM), 4 μl of nuclease-free water, and 10 μl of extracted RNA as template. Positive and negative controls were also run for each plate to ensure the validity of the PCR results. 2 μL of SARS-CoV-2 genomic RNA (BEI Resources: NR-52286) with a concentration of approximately 250 genome copies per μL and 8 μL of nuclease-free water was added to the PCR reaction mixture for the positive control sample. For the negative SARS-CoV-2 and crAssphage controls, nuclease-free water was added to the PCR reaction mixture. The plate was sealed and centrifuged at low speed, then loaded into the thermocycler. The cycling program followed the TaqMan^™^ 4X Fast Virus 1-Step Master Mix manufacturer’s recommendation: 50°C for 5 min for reverse transcription, 95°C for 20 s, then 45-50 cycles of 95°C for 15 s, and 60°C for 60 s. All RT-qPCR assays were performed using a LightCycler 480 II (Roche Diagnostics) instrument, and analysis utilized the LightCycler Software, version 1.5.1.62 SP2.

### 2.4. Data analysis

Wastewater SARS-CoV-2 concentrations were calculated based on quantification cycle (Cq) values using the Roche LightCycler 2^nd^ Derivative Maximum Algorithm. A standard curve was run (r^2^= 0.985) using serial dilutions of BEI positive control SARS-CoV-2 RNA to convert PCR CT values to genome copies per mL of sample. Our wastewater extraction process is reported to have a recovery rate of approximately 89 % and a limit of detection between 100 and 1000 viral copies per mL (Strike et al., 2022; Torabi et al., 2023). The above assay characteristics were performed by extracting SARS-CoV-2 negative wastewater spiked with heat-inactivated SARS-CoV-2 RNA (NR_52286) at concentrations between 100 and 10,000 copies per mL Laboratory staff visually inspected all amplification curves to identify errors in the machine determination of Cq values. False positive and false negative values were eliminated from the data. Staff also visually estimated the Cq values in the uncommon instances when it was determined that the software Cq value was inaccurate. We describe wastewater SARS-CoV-2 concentrations as the arithmetic average of eight aliquots (or the number of aliquots with valid results) from a given sample in units of genome copies per milliliter of wastewater (gc/mL). Similarly, we describe wastewater crAssphage concentrations as the arithmetic average of two aliquots from a given sample. It is nearly impossible to calculate the absorption, adsorption, and desorption kinetics of wastewater on a cotton passive sampler in a real-world setting. We chose to calculate SARS-CoV-2 gc/mL of squeezed eluate from the sampling device for comparison against the composite sampling method. We acknowledge that it is difficult to determine the total wastewater volume sampled through a passive sampler but based on our preliminary parallel WWTP data we found that three passive sampling devices deployed at different locations along the influent WWTP stream returned similar SARS-CoV-2 and crAssphage capture (data not shown). Additionally, sampling father upstream (such as directly outside of a LTCF) may prehomogeneous wastewater. As the CDC has done with the NWSS (Centers for Disease Control and Prevention, 2025), its likely more important to compare pathogen capture trends rather than absolute gc/mL values.

A Wilcoxon signed-rank test was used to compare the mean nucleic acid concentration values of passive and composite samples. A McNemar’s test was performed to assess the impact of the sampling method on PCR detection outcomes. A simple Kappa coefficient was calculated to determine the extent to which the outcomes resulting from the two sampling methods agreed. Preliminary analysis suggested that detection performances between passive and composite sampling may differ according to analyte concentration levels. Therefore, a stepwise threshold that was incrementally increased by 10 gc/mL between 0 and 500 gc/mL was defined to enable a stratified analysis of “high” vs. “low” concentration samples. For a given detection threshold, a 2 × 2 contingency table was constructed to reflect detection outcomes at that threshold, and a McNemar’s test was performed on the adjusted contingency table. A p-value less than or equal to 0.05 was interpreted as a statistically significant difference in outcome between the two methods.

To assess the potential for early viral detection in the LTCF setting using WBE, the reported daily new positive SARS-CoV-2 clinical test results for each LTCF were cross-correlated with SARS-CoV-2 wastewater concentrations over a range of 7 days before and after the date-matched clinical case data. Such shifts can be interpreted as SARS-CoV-2 viral detection in wastewater samples leading or lagging the detection of a new clinical case by some number of days. Kendall’s tau correlation was used in the cross-correlation analysis. The intensity of LTCF testing varied over time according to the level of SARS-CoV-2 transmission in the community and the presence of known cases in the facility. To compensate for various testing and reporting biases (e.g., limited testing results reported on weekends), a 3-day rolling average was applied to the raw reported cases data and used in cross-correlation analysis as well.

## 3. Results and discussion

### 3.1. SARS-CoV-2 detection outcomes by method

**Fig. 2.**
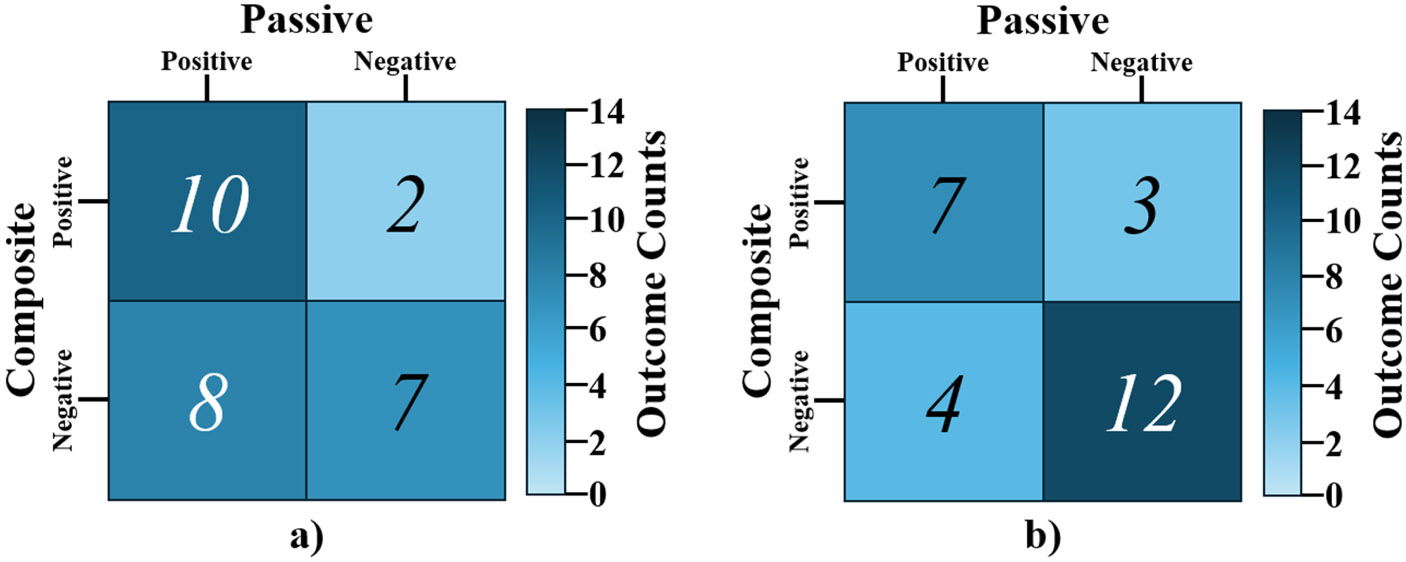
Contingency tables showing the SARS-CoV-2 detection outcomes for (a) LTCF 1 (*n* = 27), and (b) LTCF 2 (*n* = 26).

Contingency tables containing SARS-CoV-2 detection outcomes at LTCFs for the passive and composite sampling methods are shown as Fig. 2. A total of 29 positive signals were detected when using passive sampling compared to 22 positive signals when using composite sampling. The proportion of positive samples was not statistically different between the sampling methods (*p* = 0.146), indicating that the passive sampling method yielded similar detection outcomes to those of composite sampling. The degree of agreement between the SARS-CoV-2 detection outcomes obtained by the two methods was found to be fair (κ = 0.369). Taken together, results support the conclusion that passive sampling may serve as an appropriate alternative sampling method for detecting SARS-CoV-2 in wastewater when composite sampling is not feasible and absolute quantification is not necessary. We chose to forgo SARS-CoV-2 and clinical data normalization through crAssphage qPCR results, as this normalization did not significantly improve our clinical correlations at lower SARS-CoV-2 wastewater concentrations. Corchis-Scott et al. normalized passive sampled SARS-CoV-2 concentration based on the ratio of SARS-CoV-2 to pepper mild mottle virus, estimated flow rate and estimated number of infected people contributing to the effluent. However, many of these metrics are impossible to accurately estimate in LTCFs where shedding and flow rates vary dramatically throughout the day (Corchis-Scott et al., 2021). Therefore, we assume that the squeezed eluent volume is at an averaged SARS-CoV-2 concentration over the testing period, similar to how a composite sampler normalizes the bulk sampled volume over a period of time. Further studies are needed to measure the dynamic binding and elution mechanics of enveloped viruses onto passive samplers for more quantitative comparisons with conventional sampling methods.

**Fig. 3.**
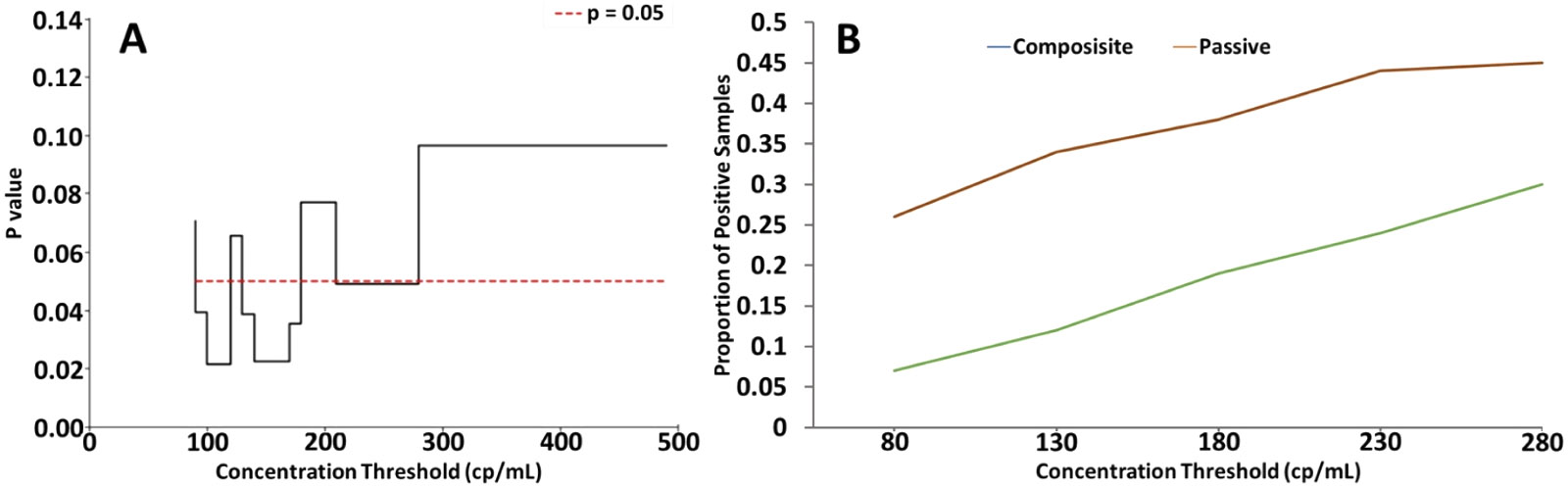
Passive sample p value advantage at lower concentration thresholds (A), and proportion of positive wastewater samples for composite and passive sampling modalities across concentration thresholds (B).

As shown in Fig. 2, there were more occasions in which SARS-CoV-2 RNA was only detected with passive sampling than with composite sampling. These discordant outcomes primarily occurred when there were low levels of SARS-CoV-2 in the source wastewater, which suggests a potential difference in sampling performance at low analyte levels. This may be due to passive sampling preconcentrating wastewater, effectively lowering the limit of detection of SARS-CoV-2 wastewater analysis in a real-world application. To illustrate this effect, we performed a series of statistical agreement tests with varying detection thresholds of the passive and composite sample SARS-CoV-2 concentration data. Specifically, we considered subsets of data that were limited to samples measuring below a threshold that was modulated from 500 gc/mL down to 0 gc/mL (i.e., these analyses only considered the “low-end” of the data). Fig. 3 shows the significance values for the low-end filter threshold analysis, where statistically significant values (i. e., where passive sampling offers a significant sensitivity advantage) are limited to concentrations below 180 gc/mL. It also should be noted that the McNemar’s test results are invalid below 80 gc/mL due to insufficient sample size (i.e., the number of discordant outcomes in the subset of interest is <6), and these values are excluded from Fig. 3. Previous comparison studies primarily focus on composite and passive sampling at either LTCFs or WWTPs (Wilson et al., 2022; Corchis-Scott et al., 2021). To our knowledge this is the first concentration-stratified comparative analysis of passive and composite WBE at both scales LTCFs and WWTPs. Our results suggest potential differences in analytical sensitivity that are dependent on the scale of wastewater sources. Table 1 shows the proportion of positive samples by sampling method for thresholds above 80 gc/mL. At a threshold between 80 and 180 gc/mL, the McNemar’s test generally reports a significant dissimilarity in the detection outcomes between the two sampling methods, indicating that passive sampling is statistically more likely to yield a positive detection at low concentrations, but this advantage vanishes for samples with higher concentrations of virus.

### 3.2. Effect of facility type on sampling performance

To investigate potential differences in sampling performance by facility, we deployed passive and composite samplers at a WWTP and compared the SARS-CoV-2 wastewater signals. Given the larger flow cross-section, three passive samplers were placed at three varying depths at the testing location in the channel to assess the swab placement on performance. The mean SARS-CoV-2 concentration detected does not significantly vary with the placement of the Moore swab (*p* = 0.4460), suggesting that the wastewater matrix in a WWTP facility is relatively homogeneous in composition at a given channel location. When comparing the average WWTP SARS-CoV-2 concentration by sampling method, passive sampling, averaged across positional replicates and all SARS-CoV-2 concentrations, outperforms composite sampling (*p* = 0.0156), yielding, in some cases, more than a 5-fold average increase in detected SARS concentration (Table 2). This result agrees with those reported in Rafiee et al. (2021) and supports our hypothesis that intrinsic differences in wastewater characteristics by facility type and level may affect the performance of passive sampling (Rafiee et al., 2021).

Differences in wastewater matrix due to the population size could potentially explain the disparity of results by facility type. The larger population, as well as possible homogenization in the sewage pipe network due to flow dynamics and travel length, could potentially produce well-homogenized wastewater with lower standard deviations, and thus a given sample would more likely reflect pathogen prevalence within the population. In contrast, wastewater from a smaller facility, with lower flow rates and shorter pipe lengths, may be more heterogeneous and therefore more susceptible to sampling error (i.e., the sample is not as representative of the population). Additionally, wastewater that reflects a small population (like the LTCFs selected for our study) may be subject to more variability in composition and relative analyte to background concentration ratio due to facility use and operations (e.g., higher use of disinfectants, differences in laundry load across different LTCFs), which could affect RNA signal obtained from wastewater.

### 3.3. Effect of sample method on SARS-CoV-2 concentration

Descriptive statistics of the wastewater genomic concentrations of SARS-CoV-2 virus are presented in Table 3. At LTCF 1, SARS-CoV-2 concentrations via passive sampling were significantly greater than those via composite sampling (*p* = 0.011) while differences in SARS-CoV-2 concentration were statistically insignificant at LTCF 2. Interestingly, the advantage in concentration of passive sampling seen at LTCF 1 was not present at LTCF 2. When comparing across locations, the overall difference in SARS-CoV-2 genomic concentrations favored passive sampling over composite sampling, although like LTCF 2, it was not statistically significant (*p* = 0.081). Approximately half of the samples tested during this period were not PCR detectable for SARS-CoV-2.

Overall, crAssphage concentrations were significantly higher in passive samples compared to the composite samples (5.66×10^5^ vs 2.21×10^7^ gc/mL, *p* < 0.001, data not shown). Significantly higher crAssphage concentrations were observed in passive samples irrespective of sampling site. Since the passive samplers act as size-selective filters in sample collection (Rafiee et al., 2021; Cha et al., 2023), target analytes associated with solids such as fecal material may be more highly represented in such samples. Our findings that crAssphage is significantly concentrated in all passive samples while SARS-CoV-2 virus is only concentrated in some passive samples suggests that passive sampling may preferentially concentrate the solid fraction of wastewater as it flows across the sampling device (Dehghan Banadaki et al., 2024). There is evidence that partitioning behavior for viruses in wastewater varies by encapsulation structure (Ye et al., 2016) and it is thought that SARS-CoV-2 is present in both fractions as whole virus or lysed genetic material. The filtering behavior of the passive sampling results in a relative abundance of pathogens in a sample that is biased in favor of targets which have strong association with solids in wastewater. Normalization of the passive sampling dataset was attempted through paired crAssphage measurements but was ultimately abandoned due to a marked decrease in post-normalization clinical correlation. This may be due to passive sampling methods preferentially concentrating the solid fraction in wastewater where crAssphage is thought to be more prevalent (Dehghan Banadaki et al., 2024) and SARS-CoV-2 genome fragments existing in both fractions to a more varied degree. Moreover, the choice of passive sampling material has been shown to impact the relative abundance of pathogens represented in a particular sample (Jones et al., 2022). Filter-based passive samplers may serve to concentrate solids-associated biomarkers in highly dilute samples, thereby enhancing analytical sensitivity of downstream detection. However, such samples would not reflect the true relative abundance of pathogens in the sample source, and the original abundances would be difficult to determine without exact knowledge of the volume sampled by the passive sampler and the partitioning kinetics of the pathogens of interest (Breadner et al., 2023). This bias could have a significant impact on the quantitative interpretation of downstream analyses.

### 3.4. SARS concentration correlation with reported clinical cases

**Fig. 4.**
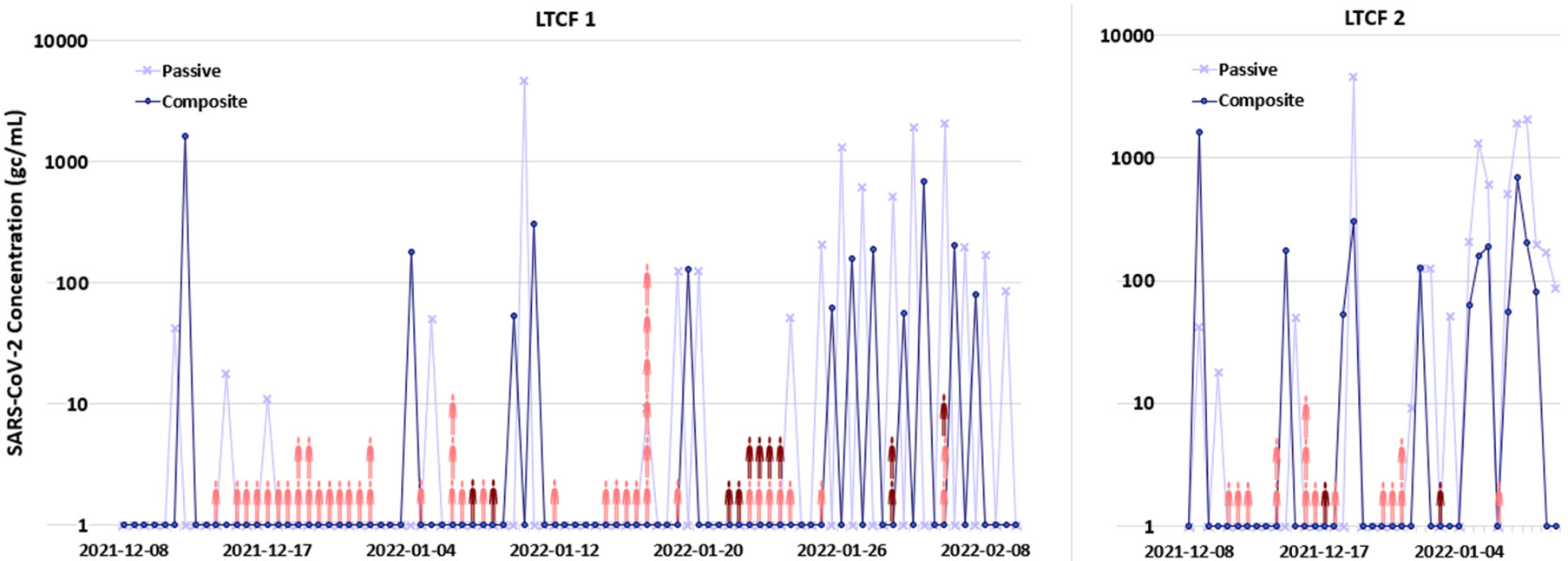
Clinical case count (single clinical case denoted by individual person icons where light red represents employee and dark red represents resident clinical data.) and logarithmic scale SARS-CoV-2 wastewater concentration at both LTCFs.

**Fig. 5.**
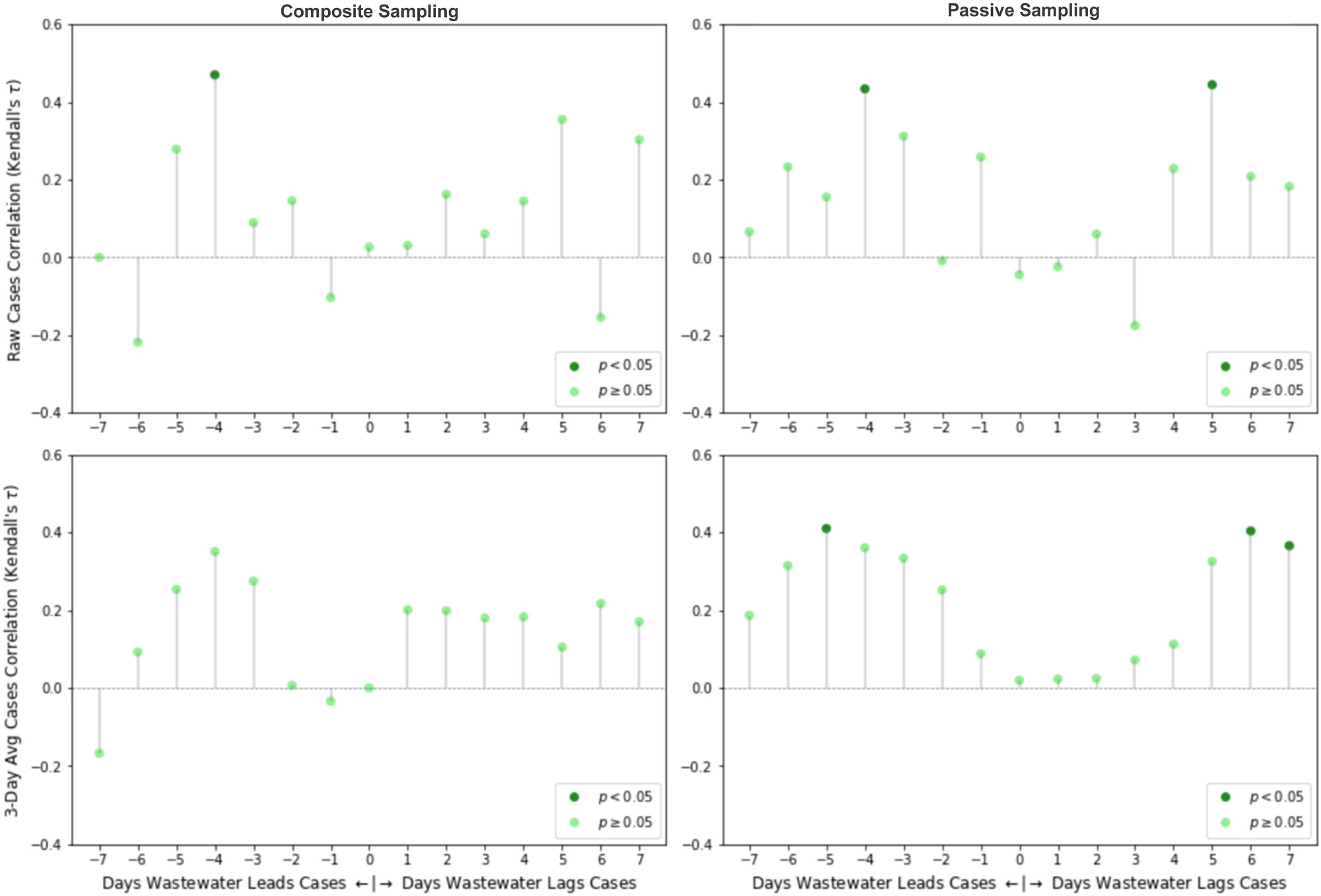
Kendall’s tau correlations between clinical cases and SARS detection outcomes at LTCF 2. Row 1: the cross-correlations between SARS-CoV-2 concentrations and unadjusted clinical cases by sampling method. Row 2: cross-correlations between SARS-CoV-2 concentrations and clinical cases adjusted with a 3-day rolling average. Dark green markers indicate significant correlations.

The reported SARS-CoV-2 clinical cases for each LTCF are shown in Fig. 4. There were 32 and 20 reported new cases at LTCFs 1 and 2, respectively, during the testing period. The majority of reported clinical cases at each LTCF were from employees (~80 % at LTCF 1 and ~89 % at LCTF 2), rather than the residents. Due to the paucity of clinical cases in the resident cohort during this period, employee and resident clinical cases were pooled for the correlation analysis, but this may have introduced error since employee response (e.g., quarantine period) varied. A temporal cross-correlation analysis was performed to evaluate the relationship between SARS-CoV-2 concentration in wastewater and reported clinical cases. No significant correlations were found at LTCF1 between the unadjusted clinical case data and the corresponding SARS-CoV-2 wastewater concentration in either passive or composite samples. In LTCF2, both the composite and passive sampling wastewater signals significantly correlated with clinical test results four days later (i.e., indicating that wastewater signal leads clinical signal). The cross-correlation results at LTCF2 are shown in Fig. 5. To compensate for day-of-the-week bias (i.e., minimal testing on weekends), we considered a 3-day rolling average, which smoothed the correlation data, but decreased the correlation coefficients for composite sampling outside of the significance threshold. However, there remained significant correlation coefficients for passive sampling (but not composite sampling). Raw case correlations with concentrations from passive samples were of moderate strength when wastewater led cases by 4 days (τ = 0.43, *p* = 0.030) and when wastewater followed cases by 5 days (τ = 0.44, *p* = 0.016); a similar trend was observed with the smoothed clinical case data, with an additional significant correlation occurring when wastewater lags cases by 7 days. Significant correlations for wastewater lead and lag could possibly be attributed to overlap with consecutive outbreaks due to the time shift. Overall, wastewater concentrations from passive samples correlated with 3-day averaged clinical cases produced stronger correlations, including a statistically significant 5-day lead correlation with clinical SARS-CoV-2 cases.

## 4. Conclusions

In this study, we successfully compared the performance of passive and composite sampling at LTCFs. While passive sampling has been around for decades, this study highlights unique advantages (and potential obstacles) with the implantation of passive sampling at LTCFs relative to sampling at WWTPs. This work is novel in that passive and composite sampling methods are simultaneously compared with clinical data at a facility level (LTCFs) and at a large population scale (WWTPs). These comparisons are needed to better understand how future WBE surveillance should be conducted accounting for cost, complexity, and need for quantification in a real-world scenario. Furthermore, this work also compares sampling methods compared across two drastically different sources and relative analyte concentrations: (1) WWTPs where a large volume wastewater is pooled together at a city-level, and (2) LCTFs where a small volume of wastewater is sampled directly outside of the facility. Overall, the use of passive sampling does not compromise detection outcomes for SARS-CoV-2 when compared with composite sampling across the full range of concentrations in this study. However, at concentrations below 180 gc/mL, passive sampling shows a significant advantage in SARS-CoV-2 detection when compared to composite sampling. Consequently, passive sampling may be appropriate for use at facilities where relatively low analyte concentrations are expected. Concentration due to the filtering of solids by the passive sampling swab may account for this advantage, but further research on adsorption kinetics of SARS-CoV-2 at varying concentration levels is required to validate this hypothesis. Contrastingly, our passive sampling study at the WWTPs revealed an obvious advantage in mean SARS-CoV-2 concentration with passive sampling, suggesting that wastewater composition and facility-level characteristics may also factor into the comparative performance of each sampling technique. Additionally, we demonstrated significant correlations between daily reported new clinical cases and SARS concentration collected for both composite and passive sampling, with SARS-CoV-2 concentrations leading clinical cases by 4 days. Passive sampling resulted in persistent correlations with both raw and smoothed clinical case counts. However, significant correlations persisted only for passive samples after smoothing of clinical case counts, which suggests that passive sampling may be better than composite sampling for tracking trends in the presence of an analyte in the LTCF setting. Additionally, it is impossible to calculate a true analyte concentration per unit of effluent due to the unknown volume of wastewater that is concentrated from a passive sampling device. However, there may be advantages for this method in a LTCF context as absolute quantification may not be as useful as general trends or limit of detection of a target pathogen. LTCF WBE is a promising framework to surveil a vulnerable population but may be limited in impact by employee infections or hygiene protocols such as adult diaper usage.

## Figures and Tables

**Table 1:** Proportion of positive samples by sampling method for moving threshold analysis.

Threshold	80	90	100	120	130	140	150	170	180	200	210	230	250	270	280
**n Samples**	27	28	29	30	32	33	34	36	37	38	40	41	42	43	44
**Composisite**	0.07	0.07	0.07	0.1	0.12	0.12	0.15	0.17	0.19	0.21	0.23	0.24	0.26	0.28	0.3
**Passive**	0.26	0.29	0.31	0.3	0.34	0.36	0.38	0.39	0.38	0.39	0.42	0.44	0.45	0.47	0.45

**Table 2:** Wastewater concentrations (in gc/mL) at WWTP.

Week	Composite	Position 1	Passive		Average
			Position 2	Position 3	
1	223	335	574	2213	1041
2	215	1390	1328	2223	1647
3	43	523	396	358	425
4	183	447	244	180	290
5	66	220	593	362	392
6	229	413	665	416	498
7	149	444	143	40	209

**Table 3:** Descriptive statistics of LTCF SARS-CoV-2 concentrations.

Site	Sample Type	(+) Samples	Mean	Medium	Range
LTCF 1	Composite	12/27	138	0	[0,1630]
	Passive	18/27	451	52	[0,4606]
LTCF 2	Composite	10/26	165	0	[0,1726]
	Passive	11/26	78	0	[0,611]
Overall	Composite	22/53	151	0	[0,1726]
	Passive	29/53	268	42	[0,4606]

## Data Availability

Data will be made available on request.
